# Can MOOC Instructor Be Portrayed by Semantic Features? Using Discourse and Clustering Analysis to Identify Lecture-Style of Instructors in MOOCs

**DOI:** 10.3389/fpsyg.2021.751492

**Published:** 2021-09-14

**Authors:** Changcheng Wu, Junyi Li, Ye Zhang, Chunmei Lan, Kaiji Zhou, Yingzhao Wang, Lin Lu, Xuechen Ding

**Affiliations:** ^1^Faculty of Artificial Intelligence in Education, Central China Normal University, Wuhan, China; ^2^School of Computer Science, Sichuan Normal University, Chengdu, China; ^3^College of Psychology, Sichuan Normal University, Chengdu, China; ^4^School of Life Science and Technology, University of Electronic Science and Technology of China, Chengdu, China; ^5^Xiaoping Executive Leadership Academy, Guangan, China; ^6^Department of Psychology, Shanghai Normal University, Shanghai, China; ^7^The Research Base of Online Education for Shanghai Middle and Primary Schools, Shanghai, China

**Keywords:** semantic features, lecture style, MOOCs, LIWC, Coh-Metrix

## Abstract

Nowadays, most courses in massive open online course (MOOC) platforms are xMOOCs, which are based on the traditional instruction-driven principle. Course lecture is still the key component of the course. Thus, analyzing lectures of the instructors of xMOOCs would be helpful to evaluate the course quality and provide feedback to instructors and researchers. The current study aimed to portray the lecture styles of instructors in MOOCs from the perspective of natural language processing. Specifically, 129 course transcripts were downloaded from two major MOOC platforms. Two semantic analysis tools (linguistic inquiry and word count and Coh-Metrix) were used to extract semantic features including self-reference, tone, effect, cognitive words, cohesion, complex words, and sentence length. On the basis of the comments of students, course video review, and the results of cluster analysis, we found four different lecture styles: “perfect,” “communicative,” “balanced,” and “serious.” Significant differences were found between the different lecture styles within different disciplines for notes taking, discussion posts, and overall course satisfaction. Future studies could use fine-grained log data to verify the results of our study and explore how to use the results of natural language processing to improve the lecture of instructors in both MOOCs and traditional classes.

## Introduction

Nowadays, multimedia learning environment, learning management system, intelligent tutoring system, and massive open online course (MOOCs) provide great opportunities to generate big data in education. Researchers from various disciplines have conducted many interesting studies in the fields of educational data mining and learning analytics. Most researchers paid much attention to analyze student data that were generated from different kinds of learning platforms (DeFalco et al., 2018; Kai et al., 2018). It helps to address personal learning demands of students and improve the quality of individualized learning. However, teaching is an important part of education as well. If the data of instructors in various teaching platforms can be fully applied, the educational data mining can provide instructors with service and further benefit students. Among various learning platforms, MOOCs has obviously become a popular way to learn for many students around the world. MOOCs provide students with opportunities to a personalized learning environment (Evans et al., 2016) and enables them participate in the cooperative learning through the discussion forum and peer evaluation. Many scholars have conducted studies about MOOCs from the perspective of the characteristics of the learners, learning effect, and course design (Khalil and Ebner, 2014; Poce, 2015; Wang and Baker, 2015), but few scholars analyzed the teaching complexity and the instructors in MOOCs (Ross et al., 2014). Teaching in traditional classes is different from the teaching in MOOCs in many aspects, such as the size of class, prior knowledge of students, and the expense of the course. Nowadays, most courses in MOOC platforms are xMOOCs, which are based on the traditional instruction-driven principle. Course lecture (i.e., course videos) is still the key component of the course. Hence, analyzing the lectures of instructors of xMOOCs would be helpful to evaluate the course quality and provide feedback to MOOCs instructors, which will further benefit learning of the students. One straightforward way is to describe large-scale MOOC lectures through natural language processing. For example, what semantic characteristics do these MOOC lectures have? Does any potential and valuable pattern exist among these semantic characteristics? Do these potential patterns associate with the learning of students? Here we define these semantic patterns that emerged from MOOC lectures as the “lecture style” of the current study. Specifically, the operational definition of lecture style is as follows: the results of cluster analysis based on the semantic features of a given MOOC video (for more details, see section Data Analysis).

When it comes to the quality of MOOCs, researchers have summarized some evaluation systems. For example, Yousef et al. (2014) conducted a large-scale survey of the learners and instructors who have the experience of MOOCs and summarized an evaluation standard of MOOCs. They found that the lectures of instructors play a vital role in the quality of MOOCs. Quality matters rubric is also a widely used evaluation rubric of online courses. This rubric makes raters mark the courses from the eight dimensions of learning objectives, namely, interactivity, usability, etc. (Matters, 2014). Integrating with the survey investigation and focus groups interview, Poce (2015) evaluates MOOC through the clarity and comprehensibility of the lecture, course design quality, etc. In the evaluation of traditional classes, the classroom instruction or course videos were often evaluated by the trained observers or experts using the mature rubric (National Board Resource Center., 2010, which is complicated and cannot avoid the subjectivity in questionnaire investigation. To address this issue, some people tried to use natural language processing to evaluate the lectures instructors of math classes (Araya et al., 2012). They extracted the semantic features from the lectures of the instructors and established several classifiers to automatically predict whether a specific category of math content (e.g., factions) or teacher practice (e.g., reasoning or immediate feedback) was covered by instructors. The results of the classifiers were compared with the experts who were invited to rate the course videos of math classes. They found that the agreements between classifiers and the raters were satisfactory. This may be a new method to evaluate the course quality. It inspires us to evaluate the lectures of the instructors in MOOCs by using natural language processing, and explore the effects of different lecture styles on the learning of students.

In their study, the linguistic inquiry and word count (LIWC) was used to count word categories related to mathematics content and teacher practice (Araya et al., 2012). With the assumption of the words people use in daily life reflect who they are and the social relationships they are in, Pennebaker et al. (2001) developed LIWC, which mainly focus on analyzing the language people use from the perspective of word frequency. Psychologists have conducted many studies in different fields by using LIWC. For example, Rude et al. (2004) found participants who are experiencing physical and emotional pain tend to have their attention drawn to themselves and subsequently use more first-person singular pronouns. Gunsch et al. (2000) found that more self-references (e.g., “I”) were present in positive political advertisements compared with mixed and negative political advertisements, whereas more other-references (e.g., “she”) were present in negative advertisements compared with positive and mixed advertisements. Researchers also applied LIWC in the field of education; Pennebaker et al. (2014) analyzed more than 50,000 essays from 25,000 students and found that word use was related to the grades of students over all 4 years of college. Robinson et al. (2013) tested whether differences in the use of linguistic categories in written self-introductions at the start of the semester predicted final course performance at the end of the semester, and the results supported their hypothesis. Based on these empirical studies, it is reasonable to use LIWC to analyze the different language use of the lectures of instructors in MOOCs.

Although LIWC is a powerful transparent text analysis program that counts words in psychologically meaningful categories, deeper discourse characteristics are still needed to analyze the lectures in MOOCs. Researchers in the field of discourse analysis proposed a multilevel theoretical framework for discourse processing (Graesser et al., 2011; Dowell et al., 2016). They identified six levels from the shallower to the deeper, including words, syntax, explicit textbase, situation model, discourse genre and rhetorical structure, and pragmatic communication. Our study relates at least to the first three levels of this theoretical framework. The first two levels (i.e., words and syntax) were addressed by LIWC. The third level in our study is textbase, which contains explicit ideas in the text that preserve the meaning. The basic units of meaning in the textbase is proposition. Proposition includes a predicate and one or more arguments. Cohesion is considered an important theoretical construct that measures the overlap between propositions in the textbase. It provides linguistic clues to make connections between an adjacent pair of sentences (Atapattu and Falkner, 2018). Higher level of cohesion in text has been found to facilitate comprehension for many readers (Gernsbacher, 1990) and is particularly important to low-knowledge readers (McNamara, 2001). When there is a lack of cohesion, an idea, relationship, or event must often be inferred by the leaner (McNamara et al., 2010). Learners with low prior knowledge lack sufficient ability generate the inferences needed to meaningfully connect constituents in low cohesion texts (O'reilly and McNamara, 2007). Cohesion is important to the lectures in MOOCs as well. Just like reading comprehension, a lecture with greater cohesion may help students to connect the discourse constituents and construct coherent meanings. In fact, the coherence assumption was one of the central theoretical constructs in the constructivist theory of discourse comprehension (Graesser et al., 1994). They assumed that students routinely try to construct coherent meanings and connections among text/discourse constituents unless the text/discourse is poorly organized. Therefore, cohesion is an essential discourse feature in the present study. Coh-Metrix will be used to extract the cohesion of the lectures in MOOCs (Graesser et al., 2004; Gao et al., 2016), and one of its central purposes is to examine the role of cohesion in distinguishing text types and in predicting text difficulty. Many studies have suggested that Coh-Metrix can be used to detect subtle differences in text and discourse (McNamara et al., 2010), and it has been widely applied in the studies of education. For example, the previous study has demonstrated that the increase in cohesion can help the students with low prior knowledge to understand the meaning of texts (O'reilly and McNamara, 2007), but the increase in cohesion does not work for the students with higher prior knowledge. As a matter of fact, students with higher knowledge can benefit from low cohesion texts because they were forced to fill in the conceptual gaps in the texts and they have sufficient knowledge to do that (McNamara, 2001; O'reilly and McNamara, 2007; Dowell et al., 2016).

On the basis mentioned above, we proposed three research questions for the current study, which are as follows: (1) Can the lectures styles of MOOC instructors be portrayed by using natural language processing? (2) What are the semantic characteristics of different lecture styles in different discipline? (3) How are the lecture styles of MOOC instructors in different disciplines associated with learning engagement (e.g., discussion posts and notes taking) and course satisfaction? To address these questions, we collected 129 course transcripts from Coursera and edX (including humanities, social science, and science), and extracted the semantic features of the lectures the instructors in MOOCs by using LIWC and Coh-Metrix. Then, cluster analysis was used to detect different lecture styles of MOOC instructors. Finally, we used ANOVA to explore the effects of different lecture styles on the learning engagement of students and perception of the course, including the number of discussion posts, notes taken, and overall course satisfaction.

## Method

### Data Collection

The datasets in the current study are course-level data, which consist of two parts, namely, text data and student data. The first part of the data was collected from the two major MOOC platforms (i.e., Coursera and edX). Convenience sampling was conducted to collect a total of 129 course transcripts (in English), and each transcript includes all sessions of MOOC. These courses cover three disciplines (humanities: 24.8%, social science: 38%, science: 37.2%), and the proportion of different discipline is relatively uniform. The average number of words per course is around 100,000 words, which ensures the robustness of the analysis results.

The second part of the data (i.e., student data) was collected from MOOC College of Guokr.com, one of the largest MOOC learning communities in Mainland China. This community offers online learners a platform where they can voluntarily evaluate MOOCs and share their opinions with fellow online learners. The community also provides various learning assistance tools, including a service for learners to take notes while taking a MOOC, as well as study groups and discussion boards for individual MOOCs. We collected the student data of the 129 courses. The student data refer to the ratings and learning engagement of the student (i.e., the number of notes taken per course and the number asynchronous discussion posts per course). Student ratings involves four dimensions, which are as follows: the amount of knowledge gained, teacher participation, interest, and curriculum design. The items include “Is the course substantial and valuable?” (The amount of knowledge), “Does the teacher participate in communication or interaction?” (Teacher participation), “Is the course interesting and attractive?” (Interestingness), and “Is the structure of the curriculum reasonable and sufficient?” (Curriculum design). A 10-point Likert scale was used, and the average of these four ratings was calculated to indicate overall course satisfaction.

### Extracting Semantic Features

Linguistic inquiry and word count 2015 and Coh-Metrix were used to extract semantic features from the course transcripts of instructors in 129 MOOCs. LIWC provides texts summary information (e.g., text length, sentence length, analysis style, etc.), function words (e.g., pronouns, articles, prepositions, etc.), cognitive processes (e.g., see, hear, and feel), emotional words (e.g., positive emotions, anger, anxiety, and sadness), biological processing (e.g., body, health, sex, etc.), drive (e.g., power, affiliation, etc.), grammatical features (e.g., verbs, adjectives, quantifiers, etc.), and informal words as the first-class semantic indices. Each first-class semantic index involves several second-class and third-class indices.

To test the cohesion of the lectures, Coh-Metrix was chosen as a supplement to LIWC. We chose referential cohesion as the index of cohesion in the present study. It refers to the degree to which there is an overlap or a repetition of words or concepts across sentences, paragraphs, or the entire text. Referential cohesion was widely investigated in the psychological studies of discourse processing (McNamara et al., 2010). Previous studies have found that lexical sophistication, syntactic complexity, and cohesion were related to the quality of writing (Kyle and Crossley, 2016; Kim and Crossley, 2018). Pronouns, emotional words, and other indices of LIWC were also found to be important in the psychological studies of discourse processing (Sexton and Helmreich, 2000; Tausczik and Pennebaker, 2010; Pennebaker, 2013; Kacewicz et al., 2014). More importantly, these semantic features could be mapped to the multiple levels of the theoretical framework of discourse analysis by Graesser et al. (2011). Thus, the following semantic features were extracted in the present study:

1. Self-reference (i.e., I, me, my, and we);
2. Emotional words, including positive emotions and negative emotions (i.e., anger, anxiety, and sadness);
3. Sentence length (the number of words contained in each sentence);
4. Cognitive words (including causality, comparison, certainty, insight, and other dimensions);
5. Big words (words with more than six letters are considered as complex words or big words in English);
6. Tone (a high number is associated with a more positive, upbeat style; a low number reveals greater anxiety, sadness, or hostility);
7. Cohesion (i.e., coreference cohesion local, the proportion of adjacent sentence pairs in the text that shares a common noun argument).

### Data Analysis

As Coh-Metrix can only analyze the texts with a length <10,000 words, the transcript of each course was sliced into several fragments with a length of 8,000–9,000 words. Then we aggregated the semantic indexes of all fragments. All data preprocessing was completed in R 3.4.3 and Microsoft Excel.

Cluster analysis (cluster package, https://cran.r-project.org/web/packages/cluster/index.html) was conducted on the selected seven semantic indices to portray the lecture styles of MOOC instructors in different disciplines. We transformed all the semantic features into *Z*-score to avoid the effect of a different variable scale. Then we performed *k*-means algorithm with Euclidean distance. The *k* value was assigned with a value from 1 to 15. Due to the sensitivity of choosing the initial center points in the clustering method, 25 initial center points were set for the configuration. Subsequently, ANOVA was conducted to explore the effects of different clusters (i.e., lecture styles) within different disciplines on the number of asynchronous discussion posts, notes taken, and course satisfaction. These results would help us to understand how different lecture styles in MOOCs influence the students learning and the perception of the courses.

## Results

Descriptive statistics of student ratings and semantic features between the three disciplines have been conducted. Please see the results in Appendix. Here, we mainly focus on the results of cluster analysis and inferential statistics.

### Results of K-Mean Cluster Analysis

**Figure 1:**
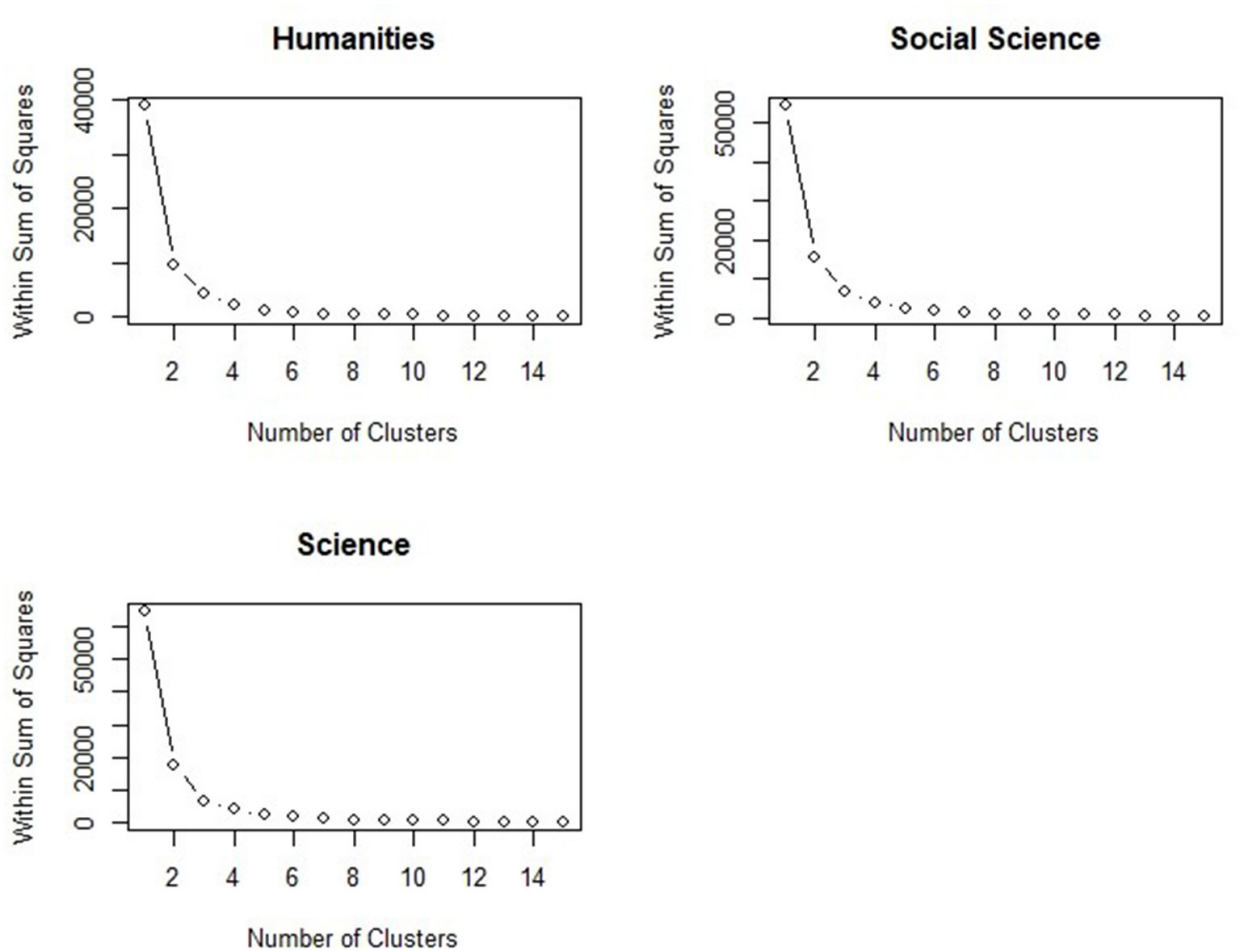
Number of clusters chosen by within sum of squares.

In all the disciplines (humanities: 38 courses, social science: 49 courses, science: 48 courses), the within sum of squares showed a significant downward trend when the number of clusters changed from one to three, and this decreased trend became slighter when the number cluster changed from four to 15 (see Figure 1). It suggested that three clusters would fit the data well in the present study. Then we conducted three K-mean cluster analysis within different disciplines (i.e., each discipline has three clusters). For humanities, 14, 10, and 8 courses were classified as Cluster A to Cluster C, respectively. For social science, 13, 14, and 22 courses were classified as Cluster D to Cluster F, respectively. As for the science, 10, 13, and 25 courses were included from Cluster G to Cluster I.

**Figure 2:**
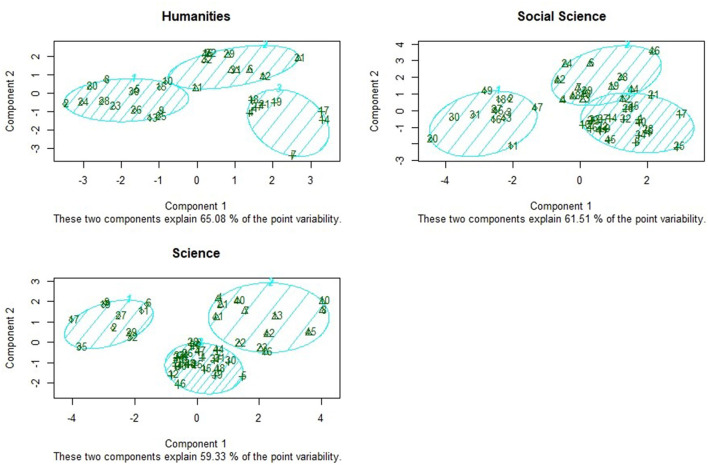
Two-dimensional representation of clusters.

Figure 2 presented a cluster amount of three classes within different disciplines. The datasets were reduced to two components (i.e., *X*-axis and -axis in Figure 1) by using principal component analysis. Except that Cluster E and Cluster F only have a small fraction of overlap in social science, the results of clustering were acceptable in general.

**Figure 3:**
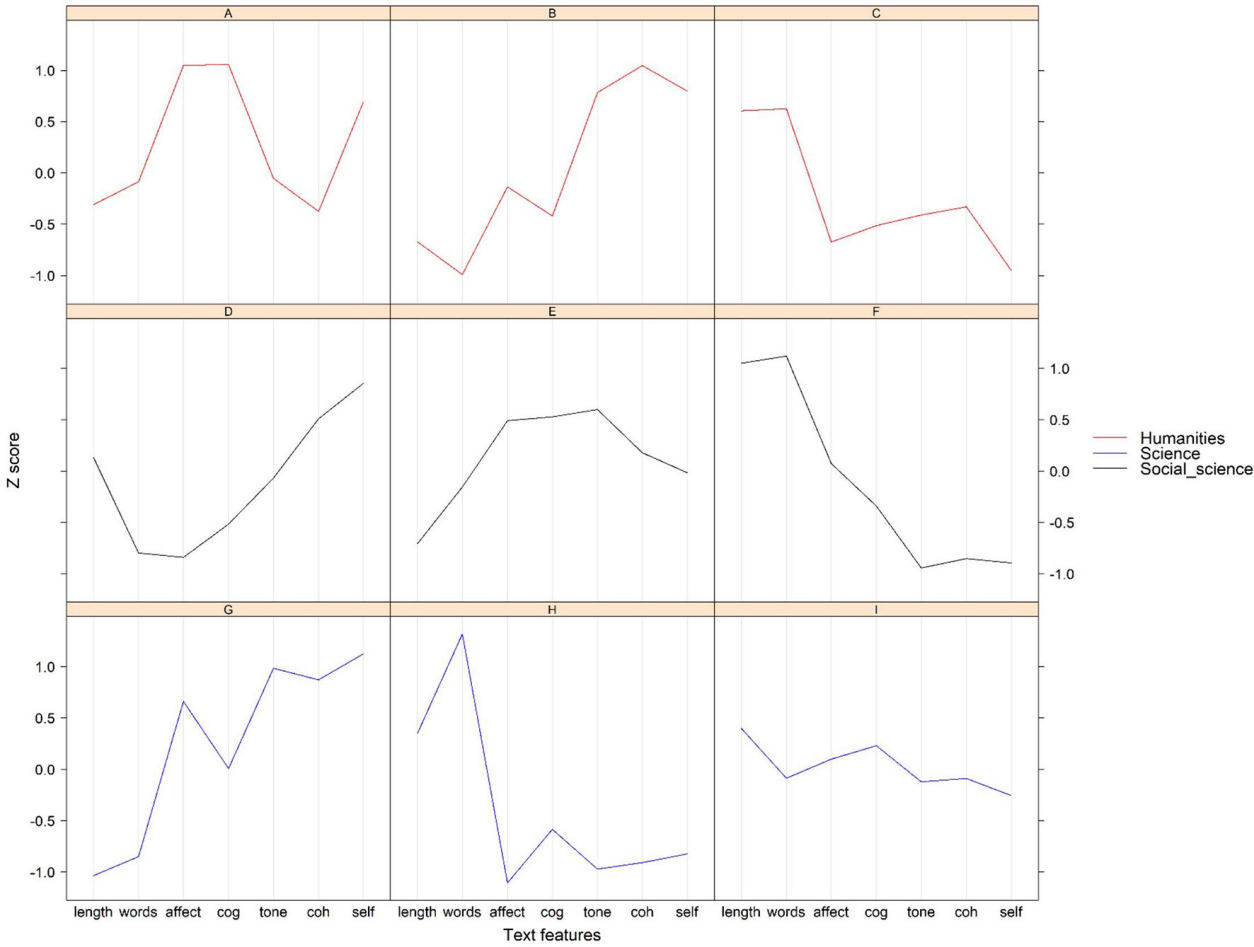
Semantic features of lecture styles in different disciplines.

We calculated the cluster means (the mean value of *z*-score for each text feature) for different disciplines. Figure 3 showed those text features of different clusters in each discipline. In humanities, the most obvious characteristic of Cluster A was that the values of affect and cognitive words were higher than the rest of the clusters, and the score of self-reference was large as well. Cluster B had the highest score of cohesion and self-reference, and the lowest score of sentence length and big words. The scores of self-reference, tone, and cognitive processing of Cluster C were the lowest among all the clusters, whereas, the score of sentence length and big words were the largest, suggesting that those instructors who were categorized into Cluster C prefer to use long sentence, complex words, and negative tone when they deliver a speech. In social science and science, both Cluster F and Cluster H have the highest sentence length and big words, and lowest tone, cohesion, and self-reference. In fact, they were similar to Cluster C. Cluster D, Cluster G, and Cluster B were similar as well, considering they all have the highest value of cohesion, self-reference, less big words and short sentence length. As for the Cluster E and Cluster I, the scores of the text features basically surrounded the mean values.

In addition, the result of Pearson correlation analysis showed that both sentence length and big words have significant negative correlation with self-reference (*r* = −0.40, *p* < 0.001; *r* = −0.61, *p* < 0.001); tone and cohesion have significant positive correlations with self-reference (*r* = 0.33, *p* < 0.001; *r* = 0.32, *p* < 0.001); and emotional words and cognitive words were not correlated with self-reference (*r* = 0.06, *p* = 0.48; *r* = 0.17, *p* = 0.06).

### Naming for the Clusters

**Figure 4:**
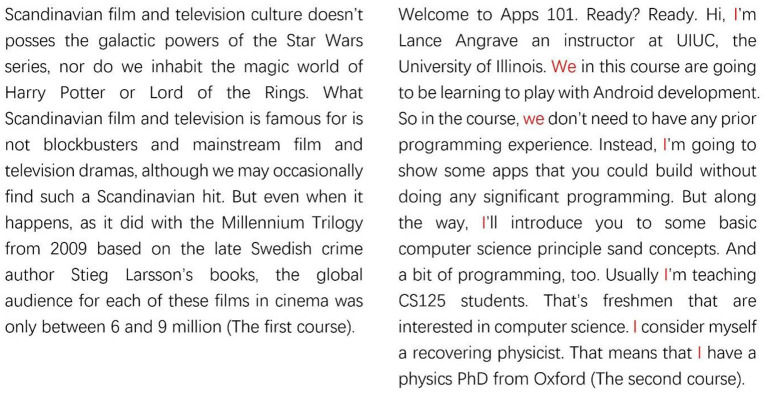
Comparison of two lecture styles (Serious vs. Communicative).

In order to present the process of naming clusters intuitively, we selected two courses from Cluster G and Cluster C as the examples. The first course belongs to Cluster G. The second course belongs to Cluster C. Figure 4 presents the beginning of the two courses. It can be seen clearly that the self-reference of the first course (at the left hand side) was low, and there were three long sentences and many complex vocabularies at the beginning of the course; whereas the second course (at the right hand side) used many self-reference words, and the sentences in this course were easy to understand. The comments from the students of two courses were also consistent with data analysis results. The following presents some of these comments:

Student A: “Some instructors in this course are serious, some are cute, and the most impressive lesson is an instructor with curly hair took us to local movie studio. The majority of the time we just watched teachers read their slides.” (Cluster C)

Student B: “…the lecture of professor is really old-fashioned, but I think it is funny to some extents…” (Cluster C)

Student C: “I feel this course may only suitable for small crowd of people, especially for the artistic youth. Although the content of course is really abundant, the lecture style of teachers is too monotonous, which makes us easy to fall asleep during watching those videos.” (Cluster C)

Student D: “The instructor has a lovely English accent, and the curriculum is reasonable. It is friendly for the beginners.” (Cluster G)

Student E: “The content is not boring, and it is easy to understand, the instructor is interesting as well. The length of the course video is suitable.” (Cluster G)

Student F: “The instructor is approachable and humorous, I like him very much!” (Cluster G)

On the basis of cluster analysis, course video reviews, and student comments, we named the four clusters in the present study as follows: perfect (Cluster A), balanced (Cluster E and I), communicative (Cluster B, D, and G), and serious (Cluster C, F, and H).

### The Results of ANOVA

An ANOVA was conducted with asynchronous discussion, notes taken, and overall course satisfaction as dependent variable, and the lecture styles within each discipline as independent variables and course popularity (i.e., the number of learners who followed the course) as the covariates. Table 1 presented the results of ANOVA and *post-hoc* test. The results of ANOVA showed that there were significant differences of asynchronous discussion (*F* = 11.32, *p* = 0.002, η^2^ = 0.28) and notes (*F* = 11.61, *p* = 0.000, η^2^ = 0.30) for humanities among the three lecture styles. Only significant difference of notes (*F* = 22.13, *p* = 0.000, η^2^ = 0.20) between the different lecture styles was found in social science. As for the science, significant differences of notes (*F* = 5.42, *p* = 0.008, η^2^ = 0.20), discussion (*F* = 4.50, *p* = 0.016, η^2^ = 0.13), and course satisfaction (*F* = 3.59, *p* = 0.035, η^2^ = 0.13) between the three lecture styles were found. Furthermore, we conducted *post-hoc* analysis by using TukeyHSD test, and the results of *post-hoc* test were presented in Table 2.

**Table 1:** The differences of notes, discussions, and course satisfaction between different clusters within the three disciplines (the followers per course was controlled as covariates).

**Groups**	***F***	***p***	***η*** ^**2**^	***M***	***SD***
Humanities: notes	11.32	0.002^**^	0.28		
Cluster A				77.40	46.17
Cluster B				59.00	26.36
Cluster C				24.57	23.82
Humanities: discussions	11.61	0.000^***^	0.30		
Cluster A				42.80	20.94
Cluster B				48.50	16.32
Cluster C				18.29	5.01
Humanities: satisfaction	3.03	0.064	0.13		
Cluster A				8.66	0.56
Cluster B				8.89	0.49
Cluster C				8.06	0.74
Social science: notes	22.13	0.000^***^	0.20		
Cluster D				24.23	22.19
Cluster E				64.59	53.69
Cluster F				69.15	43.41
Social science: discussions	2.36	0.131	0.06		
Cluster D				25.21	38.21
Cluster E				65.09	76.71
Cluster F				32.23	29.35
Social science: satisfaction	2.63	0.111	0.20		
Cluster D				8.07	0.91
Cluster E				8.76	0.30
Cluster F				8.33	0.68
Science: notes	5.42	0.008^**^	0.20		
Cluster G				23.15	18.60
Cluster H				45.20	28.35
Cluster I				49.88	30.71
Science: discussions	4.50	0.016^*^	0.13		
Cluster G				39.54	40.74
Cluster H				11.40	8.98
Cluster I				47.52	34.29
Science: satisfaction	3.59	0.035^*^	0.13		
Cluster G				9.00	0.32
Cluster H				8.04	0.60
Cluster I				8.29	1.04

*“Perfect”: Cluster A; “Communicative”: Cluster B, Cluster C, and Cluster G; “Serious”: Cluster C, Cluster F, and Cluster H; “Balanced”: Cluster E and Cluster I*.

* *p < 0.05;*

** *p < 0.01;*

*** *p < 0.001*.

**Table 2:** The results of multiple comparisons by using TukeyHSD test.

**Comparisons**	**Mean difference**	**95% CI of mean difference**	***p***
**Humanities notes**			
Cluster B - Cluster A	−18.40	−56.96 ~ 20.16	0.475
Cluster C - Cluster A	−52.83	−86.49 ~−19.17	0.002^**^
Cluster C - Cluster B	−34.43	−70.46 ~ 1.60	0.063
**Humanities: discussions**			
Cluster B - Cluster A	5.70	−11.34 ~ 27.74	0.69
Cluster C - Cluster A	−24.51	−39.39 ~−9.64	0.001^**^
Cluster C - Cluster B	−30.21	−46.14 ~−14.29	0.000^***^
**Humanities: satisfaction**			
Cluster B - Cluster A	0.23	−0.52 ~ 0.97	0.733
Cluster C - Cluster A	−0.60	−1.25 ~ 0.05	0.074
Cluster C - Cluster B	−0.83	−1.52 ~−0.13	0.017^*^
**Social science: notes**			
Cluster E - Cluster D	40.16	3.63 ~ 76.69	0.028^*^
Cluster F - Cluster D	44.73	3.57 ~ 85.88	0.030^*^
Cluster F - Cluster E	4.56	−32.82 ~ 41.94	0.953
**Social science: discussions**			
Cluster E - Cluster D	39.88	−7.86 ~ 87.61	0.118
Cluster F - Cluster D	7.02	−46.76 ~ 60.79	0.947
Cluster F - Cluster E	−32.86	−81.70 ~ 15.98	0.244
**Social science: satisfaction**			
Cluster E - Cluster D	0.69	0.17 ~ 1.21	0.007^**^
Cluster F - Cluster D	0.26	−0.33 ~ 0.85	0.537
Cluster F - Cluster E	−0.43	−0.96 ~ 0.10	0.137
**Science: notes**			
Cluster H - Cluster G	22.05	−5.98 ~ 50.07	0.149
Cluster I - Cluster G	26.73	3.94 ~ 49.51	0.018^*^
Cluster I - Cluster H	4.68	−20.25 ~ 29.61	0.892
**Science: discussions**			
Cluster H - Cluster G	−28.14	−61.73 ~ 5.45	0.117
Cluster I - Cluster G	7.98	−19.33 ~ 35.29	0.760
Cluster I - Cluster H	36.12	6.24 ~ 66.00	0.014^*^
**Science: satisfaction**			
Cluster H - Cluster G	−0.96	−1.80 ~−0.12	0.022^*^
Cluster I - Cluster G	−0.71	−1.39 ~−0.02	0.041^*^
Cluster I - Cluster H	0.25	−0.50 ~ 1.00	0.696

*“Perfect”: Cluster A; “Communicative”: Cluster B, Cluster C, and Cluster G; “Serious”: Cluster C, Cluster F, and Cluster H; “Balanced”: Cluster E and Cluster I*.

* *p < 0.05;*

** *p < 0.01;*

*** *p < 0.001*.

## Discussion

In summary, the present study extracted the semantic features of 129 MOOC transcripts and found four lecture styles (i.e., perfect, communicative, serious, and balanced). Specifically, “perfect” (Cluster A), “communicative” (Cluster B), and “serious” (Cluster C) lecture styles were found in humanities. As for the social science and science, three lecture styles emerged from these courses, namely, “communicative” (Cluster D and G), “balanced” (Cluster E and I), and “serious” (Cluster F and H). Then we collected student rating data from one of the largest MOOC learning communities in Mainland China and attempted to figure out how these lecture styles influence the learning of students. The results of ANOVA and *post-hoc* analysis indicated that learning engagement and course satisfaction were significantly different between different lecture styles within each discipline.

### Different Lecture Styles in MOOCs

The results of cluster analysis suggested that it is possible to portray MOOC instructors by using natural language processing, which answered research question 1 in the present study. There was almost no overlapping fraction in Figure 3 in any discipline, indicating the results of clustering were quite acceptable. The four types of lecture styles of MOOCs had distinctive characteristics. Similar to our results, recent studies have revealed that linguistic characteristics of texts vary across different genres and academic disciplines (Graesser et al., 2014; Medimorec et al., 2015). The most significant difference between the “perfect” presentation style and other styles in our study were the number of emotional words and cognitive words used by instructors. The *Z* scores of emotional words and cognitive words of “perfect” style were larger than 1, whereas those of the other types were <0.5. The usage of more cognitive words represents more cognitive processing (including causality, comparison, certainty, and insight) in teaching, which may benefit student learning. For example, researchers found learners read text more quickly when two-clause sentences are connected with a causal word/phrase compared with text in which a connective is neutral (Cain and Nash, 2011). Atapattu and Falkner (2018) suggested that the causal connectives in the academic discourse might improve discourse processing of the learners. Previous studies also found a moderate correlation between the cognitive activation in classroom instruction and the learning achievement (Hugener et al., 2009). Meanwhile, higher-order thinking and understanding are dependent on a high quality of cognitive learning activities in teaching (Hugener et al., 2009). High use of emotional words in the “perfect” lecture style represents an emotional speech to some extent. Researchers have found that positive emotions favor the activation of cognitive resources, which fosters task-related learning processes (Ainley et al., 2005) and metacognition (Artino and Jones, 2012). These evidences explain why we named this type of lecture as “perfect.”

As for the other lecture styles, the instructors who have “serious” style rarely used self-reference words, probably because they only focused on the presentation of course materials and relatively ignored the existence of students when they delivered their speech in MOOCs. For example, they barely introduced themselves in their speech, and rarely used “we” to establish potential connection with students. Since we have found a positive correlation between use of first-person and cohesion, it is not surprising that the cohesion of “serious” style was almost the lowest. Two of the most notable features of “serious” courses were complex words and long sentence, and we found significant negative correlations between the two features and self-reference. Instructors who have “serious” lecture style probably prefer to use written language in their lecture (lowest cohesion, lowest self-reference, most big words, and many long sentences) rather than oral language. Also, the score of big words and sentence length were almost the largest in any discipline, suggesting that the effect of the number of terminologies in different disciplines on lecture style was not as important as we thought. Previous study has found that the most common mistake instructors make is the lack of engagement during the teaching, which will make the lecture of the instructors become tedious and students will find it hard to concentrate on the lecture content (Richards and Velasquez, 2014). Instructors who have “serious” lecture styles may probably not be able to engage students during their teaching. For example, the use of “we” and “us” suggests social interaction, which helps the students sense that they are part of a class when engaging with the MOOC video (Pennebaker et al., 2007; Atapattu and Falkner, 2018). “Serious” instructors rarely use these self-reference words in their lecture video. However, the data in the present study could not verify this hypothesis directly, and further empirical studies are still needed to compare the engagement of instructors between different lecture styles.

“Communicative” lecture style (i.e., Cluster B, D, and G) almost had the opposite semantic features when compared to “serious” style. Self-reference and cohesion of the “communicative” style were higher than other styles, whereas the scores of big words and the words in per sentence were relatively low, indicating that the speeches of these instructors may be quite colloquial. According to the comments of students, we found communicative instructors were often welcomed by students. Perhaps communicative lecture style conveys more enthusiasm. A study conducted by Guo et al. (2014) found learners engaged more with the course when instructor was speaking fast, which is similar to the communicative instructors in the present study. The researchers speculated that the fast-speaking instructors convey more energy and enthusiasm. “Serious” lecture style did not have obvious characteristics when compared with other styles. The course satisfaction of Cluster G (communicative) was significantly higher than that of Cluster I (balanced) and H (serious) in science. In general, all the semantic features of “balanced” lecture style (i.e., Cluster E and I) were located around the average level, which means that this lecture style probably does not have salient characteristics.

### Impact of Lecture Style on Course Satisfaction, Discussion, and Notes

The differences of discussion and notes between the four different lecture styles were not significant in our initial study (Li et al., 2017), because of the neglect of discipline. The present study addressed this issue and found that different lecture styles had distinct semantic features, and they also had significant effects on the overall course satisfaction. In both humanities and science, instructors with “communicative” styles were more satisfied than the others. These instructors had higher level of self-reference, cohesion, and tone, which makes them to be perceived as amiable teachers (according to the comments of students). However, the “balanced” lecture style was evaluated as more satisfactory than “communicative” and “serious” styles in social science. This is probably because of the lower level of affect words, cognitive words, and tone of the “communicative” lecture style in social science, whereas the “balanced” style had higher level of affect words, cognitive words, tone, and less complex words and long sentences.

As for the learning engagement, Guokr MOOC community provides many learning tools, including a function for learners to take notes while taking a MOOC, as well as study groups and discussion boards for individual MOOCs. Many students who are not proficient in English prefer to participate in discussion in this community because they may obtain language support from the discussion forum. Therefore, the number of discussion posts and notes taken by students in each course were viewed as indices of learning engagement. Previous study has found that teacher–student interaction has a positive effect on student learning in terms of perceived motivational and cognitive learning quality of the student (Seidel and Prenzel, 2006). Similar to their study, we found that the number of discussion posts for the “communicative” lecture style in humanities was significantly larger than “serious” lecture style. Since instructors with “communicative” style were more likely to use oral language, they probably paid more attention to teacher–student interaction, which triggered more discussion. The “balanced” lecture style in science yielded more discussion posts than the others, but there was no significant difference of discussion posts between the three lecture styles in social science. It seems like the “balanced” lecture style only works for science probably because the “balanced” lecture style in science was more likely to trigger the cognitive processing of students, considering it yielded more notes taken than the other styles.

The major MOOC platforms did not provide note-taking function for MOOC learners, and many Chinese students would take notes directly on Guokr MOOC community. Notes taken reflected the cognitive processing of course content. Researchers have found that note-taking activities benefit students in exercising their self-regulated learning skills, which is an important cognitive activity in learning (Lawanto and Santoso, 2013). Also, the benefits of note-taking activity include development of higher-order thinking skills (Hohn et al., 1989; Kobayashi, 2005), and improvement of the concentration of students (Konrad et al., 2011). We found significant differences for the number of notes taken among different lecture styles. Specifically, the number of notes taken in Cluster A (perfect) was significantly higher than that of Cluster C (serious) style in humanities. Consistent with the result of cluster analysis, the “perfect” lecture style yielded a higher level of cognitive processing (i.e., notes taking), which may help students in their learning process and successfully increase their learning achievement (Lawanto and Santoso, 2013).

Interestingly, the notes of Cluster F (serious) was significantly more than Cluster E (balanced) and D (communicative) in social science. Even though the “serious” lecture style was perceived as tedious or verbose (according to the comments of students), it still yielded the higher level of cognitive processing than the other lecture styles in social science. Also, the notes of Cluster I (balanced) was significantly more than Cluster G (communicative) in science. Although the cohesion of the “serious” and the “balanced” lecture style was low, higher knowledge learners can benefit from low cohesion, because lower cohesion forces them to generate inferences to fill in the conceptual gaps (Dowell et al., 2016). Furthermore, the “serious” lecture style MOOCs was not appreciated by the comments of students, but the other sides of the courses (e.g., reasonable curriculum design, abundant course materials, effective course assignments) might affect cognitive processing of the students as well.

### Limitations and Future Directions

Some limitations of the current study should be noted. First, we did not acquire the permission to obtain the academic performance data, specific information about student profile, and other detailed data about learning engagement (e.g., the fine-grained log data) for the 129 MOOCs. We can only use the public data from a third-party MOOC community to explore the influence of different lecture styles on course satisfaction, discussion, and notes taken. Thus, it is hard to draw the conclusions in regard to the impacts of lecture-styles. Second, as Coh-Metrix can only analyze a small text (i.e., <10,000 words), we had to slice the course transcript into several pieces and then aggregate the results of all slices. This process was time-consuming, causing our sample size (i.e., 129 courses) to be relatively small. Third, we only selected seven semantic features from over 200 features to portray MOOC instructors according to previous studies and our own teaching experience. This procedure may cause information loss; perhaps automatic feature selection is a good choice as well. Fourth, the student rating data was obtained from Chinese learners. Non-native English speakers may not have good enough language skills to evaluate English MOOCs.

Future studies should acquire more detailed data about student learning in MOOCs, especially the fine-grained log data about learning progress of the students. It will allow researchers to explore the longitudinal effects of different lecture styles on the learning (e.g., engagement, affect, performance, and self-regulated learning) of the students. It is necessary to consider the effect of moderators (e.g., demographics and teaching experience) as well, especially on how teachers with different experiences moderate the effect of lecture styles on the learning of students. In addition, tracing the changes of semantic features of new teachers and providing feedback to their lecture might be helpful to improve their presentation skills. According to the current study, it seems like a good lecture should be emotional and rational. However, it may be difficult for instructors to give emotional lectures to the camera without immediate feedback from students. Moreover, simply encouraging instructors to use more emotional and cognitive words in the lecture may make instructors feel confused and have no operability. Thus, how to use the results of natural language processing to improve lectures of instructors in MOOCs is worth exploring in the future. Finally, the analysis of MOOC lecture style can also be extended to traditional classes. It might help to improve the quality of traditional courses by analyzing the presentation recording, course video, and standardized test.

## Conclusions

The results of the current study have provided answers to our three research questions. First, the lecture styles of MOOC instructors can be well-identified by natural language processing. We found that four different lecture styles emerged from 129 MOOCs, which are as follows: “perfect,” “communicative,” “balanced,” and “serious.” Second, each lecture style in different disciplines has its unique semantic characteristics. Third, the lecture styles of MOOC instructors have significant effects on learning engagement and overall course satisfaction. However, it should be noted that it is not feasible to judge which lecture style is the best or the worst without considering the instructional contexts (e.g., discipline). And more importantly, the present study only provides initial evidence with certain drawbacks.

## Data Availability Statement

The original contributions presented in the study are included in the article/supplementary material, further inquiries can be directed to the corresponding authors.

## Author Contributions

CW and JL contributed to the study's conception and design. Material preparation and data collection were performed by YZ and CL. Data analysis was performed by KZ, LL, XD, and YW. The first draft of the manuscript was written by JL and XD. All authors commented on previous versions of the manuscript, read, and approved the final manuscript.

## Funding

This present study was supported by Institute of Psychology, CAS (No. GJ202011), Application and Development of Educational Informationization Research Center of Sichuan Province (No. JYXX20-032), and Teacher Education Research Center of Sichuan Province (No. TER2019-002).

## Conflict of Interest

The authors declare that the research was conducted in the absence of any commercial or financial relationships that could be construed as a potential conflict of interest.

## Publisher's Note

All claims expressed in this article are solely those of the authors and do not necessarily represent those of their affiliated organizations, or those of the publisher, the editors and the reviewers. Any product that may be evaluated in this article, or claim that may be made by its manufacturer, is not guaranteed or endorsed by the publisher.
